# Chairside oral prophylaxis for people with profound intellectual or multiple disabilities—a retrospective feasibility study

**DOI:** 10.1007/s00784-023-05287-6

**Published:** 2023-10-25

**Authors:** Marc Auerbacher, Lydia Gebetsberger, Reinhard Hickel, Dalia Kaisarly

**Affiliations:** Department of Conservative Dentistry and Periodontology, University Hospital, LMU Munich, Goethestr. 70, 80336 Munich, Germany

**Keywords:** Oral health, Oral hygiene, Dentistry for disabled, People with disabilities, Dental prophylaxis, General anesthesia

## Abstract

**Objectives:**

People with severe intellectual or multiple disabilities (PIMD) have been receiving dental care in a specialized unit offering special care dentistry. For most of these adult patients, the initial consultation is complaint driven. In addition, the limited ability to cooperate due to their disabilities often means that dental treatment for these patients is usually carried out under general anesthesia (GA). Chairside treatment attempts are the exception rather than the rule. This retrospective study evaluated whether consistent practice of behavioral management principles and techniques embedded in a specific dental environment enables successful dental treatment of PIMD.

**Materials and methods:**

The feasibility of chairside dental prophylaxis in PIMD (*n*=36) was analyzed: specific behavioral management techniques were applied, and professional tooth cleaning (PTC) was performed in the dental chair. Clinical data obtained from medical records and a questionnaire were analyzed.

**Results:**

All patients had severe intellectual or multiple disabilities and had previously undergone at least one dental treatment under GA. Of these patients, 55.6% never had their teeth professionally cleaned before. Applying different behavioral techniques, all patients were compliant with receiving PTC in the dental chair.

**Conclusions:**

An individualized and disability-specific treatment strategy using various noninvasive and nonpharmacological behavioral guidance techniques resulted in a higher compliance rate in PIMD, which allowed chairside PTC and reduced the need for treatment under GA.

**Clinical relevance:**

Consistent implementation of various behavioral guidance techniques and communication strategies in a supportive environment enabled all patients to receive chairside PTC and be involved in a lifelong recall program.

## Introduction

The number of people with disabilities worldwide is estimated at over 1 billion, which is approximately 16% of the total population [1, 2]. At the end of 2019, approximately 7.9 million people with severe disabilities were living in Germany, of whom 13% had an intellectual or mental disability and 9% had a cerebral disorder [3]. People with profound intellectual or multiple disabilities (PIMD) have a higher risk of caries and a higher prevalence of periodontitis than people without disabilities [4–7]. The most used index to assess caries risk is the DMF/T index (D, decayed; M, missing; F, filled; T, teeth). Epidemiological studies show significantly higher DMF/T values in PIMD [8–10], and PIMD still has more missing teeth than the general population [11].

The quality of dental care services and the frequency of dental visits are lower for people with PIMD than for those without intellectual disabilities [12–14]. A systematic review of the literature on dental health in adults with intellectual disabilities found a persistently high prevalence of oral and dental disease and an increased need for dental care in PIMD [15].

However, dental care for adults with disabilities is often only provided on a complaint-based basis. Regular dental check-ups are rare for them after the age of 18, as the responsibility for pediatric dental care often ends. Regular checkups during symptom-free intervals and routine professional tooth cleaning (PTC) do not reflect the usual regimen in PIMD, although the UN Convention on the Rights of Persons with Disabilities calls for equal dental care for this vulnerable patient group [16]. Both dentist-related and patient-related reasons lead to dental treatment under sedation or general anesthesia (GA). Whereas the success of inhalational and intravenous sedation also depends on the patients ability to cooperate, e.g., when placing the mask or injecting, general anesthesia (GA) provides more safety for challenging patients because of airway management [17]. Due to complex pre-existing conditions, GA is associated with an increased risk of morbidity and mortality [18, 19]. Moreover, the dental treatment is limited to professional tooth cleaning, restorative treatment without pulp involvement and extraction.

**Fig. 1:**
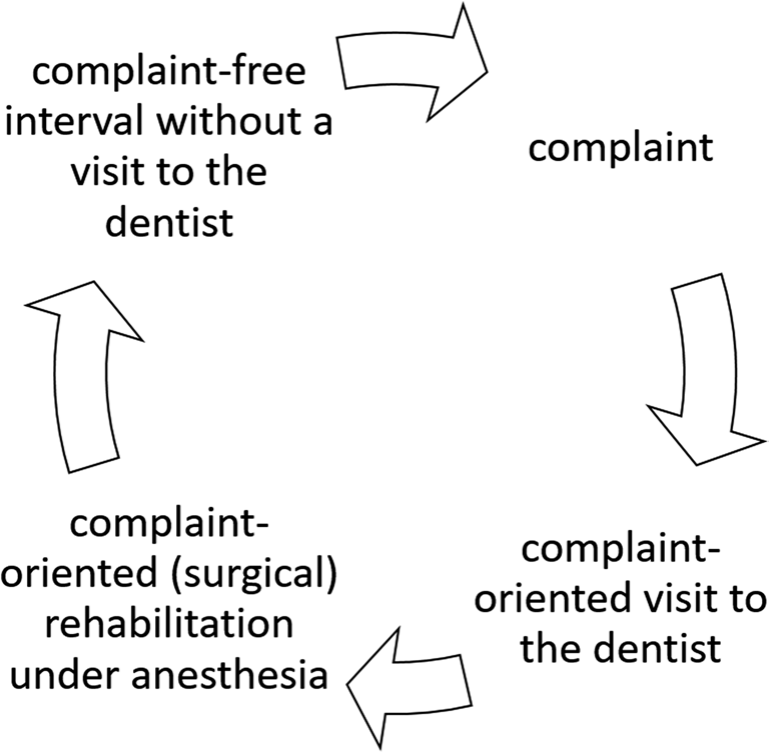
The vicious circle

Patients who underwent dental rehabilitation under GA often do not return to the dentist until the pain returns, and further treatment under GA is needed [20]. Thus, a vicious circle (Fig. 1) is created, and the symptomatic treatment under GA often means the loss of the causative tooth. Breaking this vicious circle (debonding) requires, among other things, the expertise and implementation of behavioral guidance and communication techniques in the dental treatment of PIMD. A high level of sensitivity, empathy, and motivation on the part of the dental team is also essential. The long-term goal of these confidence-building measures is also to create an individualized chairside treatment situation that will reduce the need for GA in the future.

The challenges in the dental management of PIMD are many and complex. Understanding the situation, including treatment compliance and insight, is often lacking [21, 22]. Instead, visits to the dentist are often characterized by feelings of anxiety and uncertainty. Fear is the greatest patient-related barrier to dental treatment for people with PIMD [23, 24]. Dysphagia and gagging reflexes associated with prolonged intubation, nasogastric feeding, or gastrostomy often make dental interventions in the orofacial region more difficult [25].

However, the presence of one or more of these problems hardly justifies treatment under GA, nor does a diagnosis of cognitive impairment. Many behavior guidance techniques are discussed in the literature, including tell-show-feel-do, nonverbal communication, voice position control, distraction, positive reinforcement, and desensitization. These techniques should facilitate or enable chairside dental examination and prophylaxis, even when the patient’s cooperation is severely limited [26, 27]. The British Society for Disability and Oral Health explicitly states in its clinical guidelines that GA should not be the preferred approach for PIMD treatment. In addition, alternative treatments, such as complementary psychological approaches, should be considered, and efforts should be made to provide chairside dental treatment. GA should never be used routinely for dental examinations but should be reserved for difficult and complex situations [28]. As daily oral health care can be severely limited by disability, regular recall for prophylaxis treatment or PTC is essential. Because of the importance of performing this treatment in close intervals, it is ethically questionable to do regular checkups or prophylaxis measures under GA [18].

This study aimed to investigate the feasibility of PTC compared with GA treatment in PIMD using selected behavioral guidance techniques in a specially adapted dental environment. Furthermore, dental and oral health in relation to housing (institutional or noninstitutional living) and dietary habits (sugary food and drinks) in PIMD should be evaluated. The main aim of the present study is to investigate to what extent prophylaxis treatment in the form of PTC can be performed without GA in PIMD patients with a limited ability to cooperate.

## Material and methods

### Patient characteristics

This retrospective, monoclinic study examined records and data from patients (*n*=36) with intellectual or multiple disabilities from 2015 to 2020 for first and control visits. Inclusion criteria for the current study: first, involved patients were older than 18 years. Second, in addition to a medically confirmed diagnosis of a severe intellectual disability, the patients had either a degree of disability (DoD) of 100, indicating severe disability, and/or a level of care (LoC) of at least 3, indicating severe impairment of independence according to the German health insurance system. Third, dental treatment should have taken place in the past under general anesthesia. Thus, patients younger than 18 years, having a less severe disability than that stated above, and having never had a dental treatment under general anesthesia before were excluded from the study.

The data were collected as part of the dental history and an extended medical history questionnaire and were anonymized and analyzed from the beginning of the study. The study was approved by the university ethics committee of the medical faculty (21-0202). The questionnaire included questions about age, sex, diagnosis, DoD, LoC, living situation, oral health, dental visits, diet, and smoking habits.

### Assessment of cooperation behavior

The Frankl Behavior Rating Scale (FBRS) assessed the patient’s ability to cooperate [29]. Frankl’s Behavior Rating Scores were categorized into negative (ratings 1 and 2) and positive (ratings 3 and 4) Frankl’s scores (Table 1).

**Table 1:** Frankl Behavior Rating Scale (FBRS) according to Frankl et al. (1965) [29]

Scoring	Attitude	Definition	Behavior shown during first visit
4	Definitely positive	Good relationship with the dentist, interested in the dental procedures, laughing and enjoying the situation	Shows openness and trust, allows dental procedures, not in need of special assistance
3	Positive	Acceptance of treatment, sometimes cautious, willingness to comply with the dentist, sometimes with reservation, but patient cooperates with dentist’s instructions	Familiar with the dentist’s chair able to open the mouth even for a longer period
2	Negative	Reluctant to accept treatment, uncooperative, some evidence of negative attitude, but not marked, e.g., sullen, withdrawn	Lying down on the dentist’s chair only with lots of encouragement and only for a short time, only opens mouth for a short time, allows visual examination only, requires behavioral support most of the time
1	Definitely negative	Refusal of treatment, crying violently, anxiety, or other obvious signs of extreme negativity	Refuses to lie down/sit up in the dental chair and to open his or her mouth

### Behavioral guidance techniques

The treatment concept, individually tailored to the patient’s compliance, consists of several steps. It involves the targeted use of verbal and nonverbal behavioral methods and techniques embedded in a specific dental setting. Details of the various techniques used are described in Table 2.

**Table 2:** Overview of noninvasive and nonpharmacological techniques in the dental treatment of PIMD

Technique	Goal (selection)	Practical approach	References
Patient positioning	• Muscle tone regulation • Aspiration prophylaxis	Positioning aids (cushions)	[30–34]
Voice control	• Achieve a calming and de-escalating effect • Focusing attention	A pleasant, calming, and motivating voice guides the treatment	[35, 36]
Tell-show-do (*TSD*)	• Anxiety reduction • Desensitization	Explain what you want to do (tell) Show what is involved (show) Carry out the procedure (do)	[35, 36]
Positive reinforcement	• Encourage desired behavior	Positive consequence (e.g., praise) for desired behavior	[35, 36]
Nonverbal communication	• Communication at nonlinguistic and paralinguistic levels	Communication using nonverbal signals (e.g., facial expressions, gestures, physical contact)	[37]
Plain language	• Establish a basis for communication	The rules of plain language	[38]
Distraction	• Shifting attention • Overlapping sounds	Patient distraction (verbal, visual, acoustic)	[39]
Marte Meo^®^ *(“The good face”)*	• Create a positive relationship atmosphere • Signal acceptance and impartiality	Present a “good face” (friendly, well-meaning, authentic)	[40]
Facio-oral tract therapy (*F.O.T.T.*^*®*^*)*	• Developing tolerance to touch and intervention in the orofacial area • Focus attention	Extra and intraoral stimulation through structured tactile input	[41]
Protective stabilization	• Short-term assessment and/or chair-side treatment at for motor agitation	Support the head, jaw, and/or extremities. Informed consent required	[42]

### Dental findings

Dental findings were collected and presented as dental status (DMF/T) and periodontal status (Periodontal Screening Index (PSI)).

### Professional tooth cleaning

PTC includes the removal of hard and soft dental plaque, polishing of tooth surfaces, use of an interdental brush aid, and fluoridation. In addition, relatives and caregivers were instructed in oral hygiene measures (train the trainer).

### Statistical analysis

Statistical analysis was performed using IBM SPSS Statistics (version 26.0, SPSS Inc., Chicago, IL). Quantitative variables (age, degree of disability, level of care, Franklin behavior rating scale, DMF/T, PSI) were descriptively presented as the mean and standard deviation (SD). The mean and SD of PSI were calculated using the highest score of each patient. Categorical variables (gender, diagnosis, treatment spectrum, and questions from the questionnaire) were presented as frequencies (*N*, in percent). Linear regression (DMF/T dependent variable) examined the relationship between housing situation and DMF/T and the relationship between dental care and DMF/T. The level of significance was set at *α* = 0.05.

## Results

### Patient characteristics

**Fig. 2:**
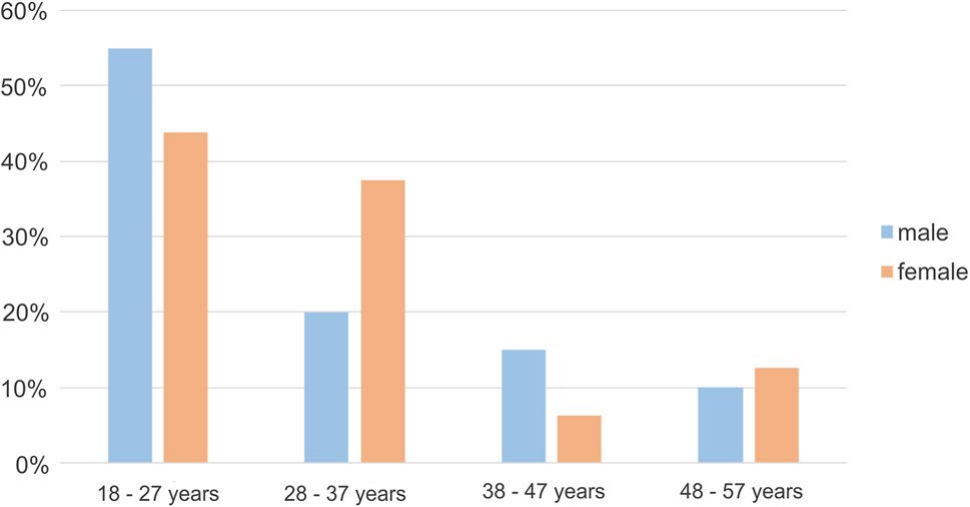
Baseline characteristics (age and gender) of the patient population with the majority of patients being young

The baseline characteristics of the patient population are shown in Table 3. Patients were over 18 years of age at the time of first contact with a mean age of 30.4 (10.1) years, 55.6% male (*n* = 20), and 44.4% female (*n* = 16); that is, most of the patients were rather young (Fig. 2).

**Table 3:** Baseline characteristics of patients with intellectual or multiple disabilities, data specification: mean and standard deviation, or frequency in %

	All (*n*= 36)
Age (years)	30.4 (10.1)
Female, *n* (%)	16 (44.4)
Degree of disability	100 (0)
Level of care	3.78 (0.7)
Diagnosis, *n* (%)	
Cognitive disabilities	11 (30.6)
Multiple disabilities	13 (36.1)
Rare diseases	12 (33.3)
Living situation, *n* (%)	
Institutional living	
Living in institutional care	12 (33.3)
Living in a nursing home	3 (8.3)
Noninstitutional living	
Living alone	1 (2.8)
Living with family	20 (55.6)
Frankl behavior rating scale	
Definitely positive	3 (8.3)
Positive	9 (25)
Negative	14 (38.9)
Definitely negative	10 (27.8)
Dental and periodontal status	
DMF/T	7.2 (6.6)
DT	1.5 (2.7)
MT	2.1 (2.9)
FT	3.7 (4.2)
PSI	3 (0.7)
Last dentist visit (years)	
≤ 1 year	23 (63.9)
> 1 year	13 (36.10)
Dental treatments, *n* (%)	
Treatment under general anesthesia	36 (100)
Professional tooth cleaning alio loco [≥ 1]	16 (44.4)
Professional tooth cleaning never before	20 (55.6)
Oral care, *n* (%)	
Assisted oral hygiene	27 (75)
Electric toothbrush	20 (55.6)
Dental care products	11 (30.6)
Frequency of tooth brushing per day	
≥ 2	21 (58.3)
< 2	15 (41.7)
Minutes of tooth brushing [min]	
≥ 2	9 (25)
< 2	22 (61.1)
Not specified	5 (13.9)
Replacement of toothbrush [weeks]	
≤ 8	17 (47.2)
> 8	18 (50)
Not specified	1 (2.8)
Frequency of sugar intake [day]	
> 1	10 (27.8)
≤ 1	26 (72.2)
Sugary beverages, *n* (%)	29 (80.6)
Smoker, *n* (%)	2 (5.6)

*DMFT* decayed, missing, and filled teeth, *PSI* periodontal screening index

All patients had an officially recognized severe disability with a DoD of 100 and a LoC of at least 3 (mean 3.78 ± 0.7) or higher. Fifty percent (*n* = 18) of the patients had a LoC of 3, and 22.2% had a LoC of 4 (*n* = 8). The highest LoC 5, which represents the most severe impairment of independence with special care needs, was therefore held by 27.8% of patients (*n* = 10).

**Fig. 3:**
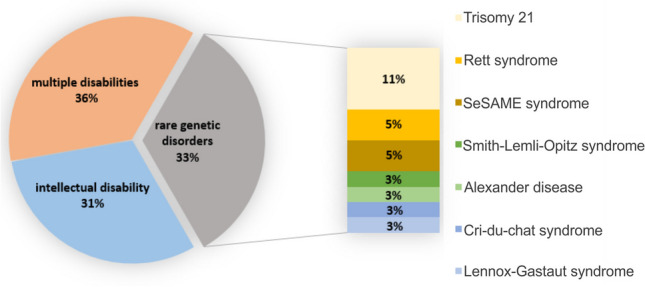
Distribution of the disease patterns (disabilities and syndromes) in the patient population

Intellectual disability was diagnosed in 30.6% (*n* = 11), multiple disabilities in 36.1% of patients (*n* = 13), and a genetic syndrome disorder in 12 patients (33.3%), as displayed in Fig. 3.

More than half of the patients (55.6%) were cared for in a family environment. Inpatient care is available for 41.6% of the patients, of whom 25% live in an assisted living group, 8.3% in a shared apartment, and the same proportion in a home. Only one patient was in an outpatient residential care setting.

All patients had previously been treated under anesthesia due to their limited ability to cooperate (100%). Of the 36 patients, 16 patients (44.4%) had previously received a PTC alio loco, of which 9 (25%) were in GA and 20 (55.6%) patients had never received a PTC.

### Assessment of cooperation behavior

According to the FBRS, 67% were uncooperative (definitely negative or negative), and 33% were cooperative (positive or definitely positive).

### Behavioral guidance techniques

To enable the treatment of PIMD in the dental chair, different techniques have been used according to each patient’s behavior (Table 2). The most used techniques for all patients and treatments were positioning, voice control, verbal and nonverbal communication, tell-show-do, and positive reinforcement. For restless and anxious patients, distraction to shift the focus has been successful. If a patient has shown signs of oral hypersensitivity, desensitization measures (such as tactile input or F.O.T.T., Table 2) have been implemented. All employees were trained to show an open, warm, and welcoming attitude toward PIMD. For a patient with limited cooperation or a previously cooperative patient who rapidly became uncooperative, protective stabilization was used. The application always required the consent of relatives or legal guardians and was in line with the American Academy of Pediatric Dentistry (AAPD) [43]. Only if the patient could not be regained by other behavioral management was this technique used to protect the patient’s safety and to facilitate the completion of the treatment.

### Dental (DMFT) and periodontal (PSI) status

The mean value of DMF/T was 7.2 (6.6), and the mean PSI was 3 (0.7). Linear regression showed no influence of the predictors on the DMF/T (*p* > 0.05) (Table 4).

**Table 4:** Linear regression, predictors of DMFT in patients with intellectual or multiple disabilities

Independent variables	Stand. beta	*p* value
Housing situation	0.327	0.051
Dental hygiene	−0.168	0.327
Dental hygiene frequency	0.072	0.675
Electric toothbrush	0.218	0.209
Exchange toothbrush/brush head	−0.171	0.325
Sweets	0.084	0.626
Sugary beverages	−0.059	0.733
Smoker	0.184	0.314

### Oral care

Oral hygiene behavior and dietary habits in PIMD were assessed (Table 3). Most of them (75%) needed help with oral hygiene and brushed their teeth or had help brushing their teeth two or more times a day (58.3%). More than half (61.1%) spent less than two minutes brushing daily. Just over half (55.6%) used an electric toothbrush, and half (50%) replaced the brush head or toothbrush after more than 8 weeks. Additional dental care products such as dental floss, interdental brushes, or mouthwashes were used by 30.6%. Regarding dietary habits, 72.2% of the patients consumed sweets less than once a day, but the majority (80.6%) consumed sugary drinks daily. Only 2 patients (5.6%) were smokers.

### Professional tooth cleaning

In all patients (100%), behavioral guidance techniques by an experienced and understanding team enabled chairside PTC to be performed. Prophylaxis using the different techniques took an average of 30–45 minutes per patient. The effort of each treatment was to remove soft and hard plaque, polish the tooth surface, and apply fluoride. Plaque and calculus were removed using ultrasonic scaler and/or hand scalers; however, in cases of diminished compliance, only the polishing brush was used with a polishing paste. Fluoridation measures were always the final step of each treatment following plaque removal.

Not every PTC could be performed the same way for every patient, as the patient’s willingness to cooperate also depended on their daily condition and could vary accordingly. In the same way, complete cleaning was only possible in some patients after gradual habituation to the treatment. To maximize the patients’ cooperation, several techniques were used to meet patients’ needs at an individual level. When patients were sufficiently cooperative (FBRS 3 and 4), PTC included removal of soft and hard deposits. This was usually done with hand scalers, but if the patient tolerated noise and had no dysphagia, the ultrasonic scaler was also used to remove plaque and calculus. Afterwards, tooth surfaces were polished by brushing with a prophylactic paste, interdental spaces were cleaned with interdental brushes or dental floss, and finally tooth surfaces were coated with fluoride varnish. Only if the patient’s compliance was not enough for all steps (FBRS 2 and 1) we used prophylaxis brushes and paste. A step-by-step approach could help some patients to tolerate hand instruments or even sound in further treatments. Fluoridation measures have always been the final step of each PTC, but always combined with prior cleaning.

## Discussion

With this study, we can show how dental prophylaxis in PIMD can be successfully performed even with poor cooperation and how individualized treatment concepts with nonpharmacological techniques can reduce the incidence or frequency of GA. All patients received dental treatment under GA during their lifetime once or more. Less than half of the patients had previously received PTC alio loco at least once, mostly under GA. Our results show that it is possible to perform chairside PTC in all patients included in this study, thus giving the patients the chance for a regular and lifelong recall and a dental home.

At the start of treatment, most patients (67%) showed little or no willingness to cooperate. These patients initially refused to lie on the dentist’s chair and open their mouths. Others spent only a short time in the dentist’s chair, allowing only a visual examination of the teeth and no instruments to be used. A smaller proportion of patients (33%) responded to the chairside treatment with openness and trust and tolerated being examined with instruments for more than a few seconds. Thus, allowing for a detailed dental diagnosis and PTC at a later appointment. This unusual behavior may lead many dentists to believe there is no possibility of treating these patients in the dental chair, and the indication for treatment under general anesthesia is made prematurely. In uncooperative patients, the absence of pain and other findings that would require immediate action is a condition for attempting treatment while awake. Therefore, the best time to start treatment trials for PIMD is during symptom-free intervals, when there is no suffering that could further increase stress and arousal levels.

Chairside treatment attempts are the exception rather than the rule. Actually, the common practice is to treat PIMD under GA which is partly due to compliance issues of the patients but also due to financial aspects in the dental offices as their treatment in the wake state would be much more time consuming with only little extra financial compensation. The literature suggests that dental treatment of disabled people is primarily performed under GA. The literature emphasizes that sedation or GA plays an important role when providing special care dentistry for people with disabilities. At the same time, it is pointed out that many practitioners are inadequately trained in the use of non-invasive and non-pharmacological techniques [27, 44–48]. This leads to PIMD patients being treated under GA even when it would be possible to do so in a routine dental setting with the necessary skills. This opinion is also shared by professional associations, including the American Academy of Pediatric Dentistry and the British Society for Disability and Oral Health. Some authors are also critical of the fact that treatment choices are made on the basis of reimbursement rather than on the basis of the best choice for the patient, since the use of time consuming behavioral guidance techniques is not covered by the insurance.

In the presence of pain and a lack of cooperation, sedation or GA is usually unavoidable to prevent serious consequences. Severe agitation due to spasticity or anxiety, which is common, may limit the duration of prophylactic treatment. This can compromise the quality of the PTC, whereas sedation or GA offers the advantage of treating patients in a relaxed or asleep state. As dental prophylaxis in PIMD should be carried out several times a year, it is not recommended to use sedation or GA all the time. Efforts should always be made to provide prophylactic treatment while the patient is awake. Each treatment attempt can help to gradually improve the compliance of PIMD and develop strategies for coping with disability-related limitations.

In our study, the plaque index was not measured as the use of plaque revelators is difficult due to limited patient cooperation. Unfortunately, we did not collect a visible plaque index form the beginning in all of our patients, so we did not include this information in the analysis. However, with the help of the PSI, statements can be made about gingivitis or the suspicion of periodontitis.

The current study is limited by its small sample size. The data came from patients with a similar catchment area and who were treated in the same facility which makes it difficult to generalize our outcome. Other studies examining dental health in PIMD showed similar DMFT values in comparable age groups [49, 50], while others have found a higher DMFT [51, 52]. This may also be due to the older age of the participants, as there are more decayed and missing teeth in PIMD with increasing age [53].

This retrospective study was designed to assess the feasibility of chairside oral prophylaxis in PIMD with the following criteria for determining success: first, performance of chairside PTC in PIMD who had previously undergone GA for dental treatment or never had PTC before and second, PTC included the removal of soft and hard deposits, polishing, and fluoride varnish application. However, future research is needed to explore the feasibility in other dental facilities with similar patients and equal conditions especially in terms of staff, time, and space.

In the current study it was unknown whether the patients had received individual prophylactic measures during childhood which is part of the standard care in the German health care system. Nevertheless, 20 (55.6%) patients in this study never have received prophylaxis before, neither as children nor as adults. We even see patients 18 years and older who have never been to a dentist before. This is dramatic since our health care system should be accessible to everyone. Sometimes parents or relatives reported that they were not able to find a dentist who is willing to perform even a regular dental checkup. In other cases, they reported feeling ashamed of going to a normal dentist with their disabled relative because they scream very loudly or show aggressive behavior in stressful situations.

Dental treatment for PIMD is time-consuming and requires more staff, special access, and space requirements. These additional costs are not usually covered by health insurance. This may be one reason why treating PIMD is unpopular and avoided by many dentists. To ensure high-quality dental care for PIMD, the costs must be covered by the health care system. Politicians and social organizations need to create a legal framework for health insurers to cover the costs. Another reason is the limited or nonexistent training in the management of PIMD during dental school. At German universities, the treatment of PIMD is taught as an elective, as it has not been part of the dental curriculum for the last 50 years. With the introduction of a new dental licensing regulation in 2021, there is a focus on patients with special health care needs. Using theoretical and practical teaching formats, the focus should also be on teaching the treatment techniques necessary for the dental treatment of PIMD. Only if people with special health care needs are part of the curriculum can there be a chance that future dentists will be willing and able to treat this vulnerable patient population in their future profession [54].

The overall risk of sedation and GA has been further reduced in recent years by safe and well-controlled anesthesia with fewer side effects and recent developments in pre- and postoperative management. However, the potential for complications following anesthesia in medically compromised patients with special needs should not be underestimated. It has been reported that aspiration occurs in approximately 3 out of every 10,000 anesthetic procedures, with vulnerable patient groups at particular risk [55]. The presence of multiple comorbidities in PIMD may also increase the risk of complications during treatment under GA [56]. For example, people with mental retardation were found to have increased blood pressure and heart rate after surgery, as well as significantly higher levels of cortisol and prolactin [57]. Patients with a rare genetic disorder that affects heart, liver, or kidney function require special observation and continuous monitoring when undergoing GA [58]. There is evidence in the literature that children or adults with Williams Syndrome have increased morbidity and mortality during GA due to cardiovascular involvement [59, 60]. However, friendliness, emotional empathy, and unusual attraction to strangers are characteristics of people with Williams Syndrome and may be useful in establishing confidence-building measures between them and the dentist [60].

The risk of developing epilepsy after GA is increased in patients with comorbidities, as is the risk of increased seizure frequency in patients with a preexisting seizure disorder [61, 62]. The time and effort required for preoperative assessment and preparation for anesthesia should not be forgotten, as this already causes high-stress levels in many patients [63]. In addition, postoperative monitoring and intensive care of PIMD are usually not feasible with low nurse staffing levels [64]. If the patient requires stationary monitoring, it may be unavoidable for a family member to stay overnight with them.

The many and varied nonpharmacological behavior management strategies that can be used to build trust and promote cooperative behavior in PIMD have received little attention in the literature. Existing studies and reports examine the efficacy of these techniques, particularly in relation to dental anxiety, and report positive long-term effects [65]. Notably, most of these studies have been conducted with anxious people who do not have intellectual disabilities. Similar studies are needed to confirm the effectiveness of these chairside behavioral techniques in PIMD so that individualized treatment approaches and strategies can be established.

The Frankl Behavior Rating Scale (FBRS), which was used in this study to assess patient cooperation during dental treatment, is very common in dentistry. It should be noted that the FBRS was originally developed for pediatric dentistry [66]. To our knowledge, there is no rating scale to assess cooperation during dental treatment in adults with intellectual disabilities. Patients’ impairments were based on the degree of disability and level of care. Cognitive ability, manual dexterity, or ability to perform activities of daily living was not assessed.

According to the criteria of evidence-based medicine, there is little systematic evidence of behavioral management techniques. The best evidence currently available comes from uncontrolled trials, individual case studies, and expert opinion, mainly in pediatric patients [67]. The limited evidence for the effectiveness of one technique over another needs to be clarified by further research in the field of dental treatment of PIMD. Most publications describe and promote the use of behavioral management techniques in the dental treatment of children and adolescents with intellectual disabilities. The present work successfully tested the use of these techniques in the dental treatment of adult patients with PIMD.

As PIMD is a very heterogeneous group, the same techniques may work differently in patients with the same type of disability. The combination of several techniques may be necessary to be successful [26]. When talking about behavioral support strategies, it is important to note that successful implementation depends on certain conditions. These include continuity and familiarity in the composition of the dental team, prior experience, and knowledge in dealing with PIMD, a time frame that includes breaks, and spatial requirements that allow good access to PIMD. Finally, it should be noted that in no other area of dentistry is the success of dental treatment as closely linked to individual willingness, motivation, personal commitment, and empathy as in the treatment of PIMD. Dental knowledge and skills are the basic requirements for the successful treatment of PIMD.

Dental treatment under GA should always be an ultima ratio indication. This is when the patient’s ability or willingness to cooperate is insufficient and all alternative techniques have not led to the desired success. The gradual adaptation of PIMD to dental treatment with the help of special methods and techniques offers the chance that PIMD will not only tolerate dental treatment better, but that cooperation can also be improved in the long term. This can be an important step toward a successful and lifelong dentist-patient relationship.

## Conclusions

An individualized and disability-specific treatment strategy using various noninvasive and nonpharmacological behavioral guidance/support techniques resulted in a high compliance rate in PIMD. Improved compliance allowed chairside prophylaxis treatment and may reduce the need for treatment under GA.

## Data Availability

Data sets analyzed in this study are not publicly available.
